# Monolayer Graphene Radiation Sensor with Backend RF Ring Oscillator Transducer

**DOI:** 10.3390/nano12030305

**Published:** 2022-01-18

**Authors:** Mohamed W. Tawfik, Abdelhameed Sharaf, Mohamed Serry

**Affiliations:** 1 Department of Mechanical Engineering, American University in Cairo, New Cairo 11835, Egypt; mwaheed@aucegypt.edu; 2 Department of Radiation Engineering, Egyptian Atomic Energy Authority, Cairo 11765, Egypt; a_sharaf@aucegypt.edu

**Keywords:** graphene, monolayer, radiation, sensor, lattice defects, gamma radiation, beta radiation, RF, ring oscillator, transducer, Raman spectroscopy, defect density, morphology, scanning electron microscope

## Abstract

This paper proposes a new graphene gamma- and beta-radiation sensor with a backend RF ring oscillator transducer employed to convert the change in the graphene resistivity due to ionizing irradiation into a frequency output. The sensor consists of a CVD monolayer of graphene grown on a copper substrate, with an RF ring oscillator readout circuit in which the percentage change in frequency is captured versus the change in radiation dose. The novel integration of the RF oscillator transducer with the graphene monolayer results in high average sensitivity to gamma irradiation up to 3.82 kΩ/kGy, which corresponds to a percentage change in frequency of 7.86% kGy^−1^ in response to cumulative gamma irradiation ranging from 0 to 1 kGy. The new approach helps to minimize background environmental effects (e.g., due to light and temperature), leading to an insignificant error in the output change in frequency of the order of 0.46% when operated in light versus dark conditions. The uncertainty in readings due to background light was analyzed, and the error in the resistance was found to be of the order of 1.34 Ω, which confirms the high stability and selectivity of the proposed sensor under different background effects. Furthermore, the evolution of the graphene’s lattice defect density due to radiation was observed using Raman spectroscopy and SEM, indicating a lattice defect density of up to 1.780 × 10^11^/cm^2^ at 1 kGy gamma radiation, confirming the increase in the graphene resistance and proving the graphene’s sensitivity. In contrast, the graphene’s defect density in response to beta radiation was 0.683 × 10^11^/cm^2^ at 3 kGy beta radiation, which is significantly lower than the gamma effects. This can be attributed to the lower p-doping effect caused by beta irradiation in ambient conditions, compared with that caused by gamma irradiation. Morphological analysis was used to verify the evolution of the microstructural defects caused by ionizing irradiation. The proposed sensor monitors the low-to-medium cumulative range of ionizing radiations ranging from 0 to 1 kGy for gamma radiation and 0 to 9 kGy for beta radiation, with high resolution and selectivity, filling the research gap in the study of graphene-based radiation sensors at low-to-medium ionizing radiation doses. This range is essential for the pharmaceutical and food industries, as it spans the minimum range for affecting human health, causing cancer and DNA damage.

## 1. Introduction

Radiation sensors are widely used to monitor radioactive activities continuously in multiple applications such as biomedical diagnostics, pharmaceutical applications, border security, agriculture, environmental applications, astrophysics, and nuclear power plants [1,2]. There are two main radiation classes: particle radiation such as alpha, beta, and proton radiations and electromagnetic radiation, including the entire electromagnetic spectrum but more harmfully, gamma radiation [3].

Beta irradiation results from particle ejection from a radioactive nucleus in one of two forms: negatively charged electrons (beta minus, an electron) or positively charged electrons (beta plus, a positron). Beta radiation can travel long distances in the air and penetrate the body by up to 3.8 cm, causing mainly skin cancer [4,5,6]. On the other hand, gamma radiation is electromagnetic radiation consisting of photons emitted by the relaxation of the daughter nucleus after radioactive decay of the unstable or parent nucleus [7]. Gamma radiation is very harmful because it is ionizing radiation, i.e., it causes the absorbing matter to emit electrons. Thus, it may interact with the nucleus of the matter, causing positron emission. This may damage DNA and cause different types of cancers in humans, including skin, bone, breast, and lung cancer and leukemia [8].

Conventional radiation detectors, e.g., gas-filled detectors and scintillators, have several limitations [9]. Gas-filled ionization chamber detectors detect high radiation doses but have low sensitivity in detecting lower radiation ranges. Thus, they are mainly used in the nuclear power industry. They have several disadvantages, such as low sensitivity for small-sized chambers and being affected by changes in humidity and barometric pressure [10]. The Geiger-Mueller (GM) detector is another gas-filled detector type, with the ability to detect low-range doses but the drawbacks of having a slower response and lower selectivity, as it is affected by background effects [9,11]. Scintillators can detect high-energy radiation emissions using a low-photon-energy response for liquid or solid material. Scintillators can also detect the distribution of radiation emissions in the environment, making them a good candidate for quality inspection and medical diagnosis applications [12]. However, they have low selectivity with limited resolution and are also affected by ionization quenching, having a lower response with a high ionization density of charged particles [13]. Furthermore, the detectors mentioned above are large, heavy, and bulky and have high power consumption, rendering them unsuitable for battery-powered sensor networks.

Solid-state detectors have gained much attention recently due to their simplicity, low cost, and high sensitivity [14]. In addition, semiconductor-based solid-state radiation sensors are widely used because they can generate numerous electron–hole pairs in response to incident radiation generated at low photon or particle energies, compared to conventional scintillators [15]. Solid-state radiation sensors and the materials used in them can be classified into two main classes: single detectors, which are mainly fabricated using silicon, germanium, or metals (e.g., platinum) and compound detectors, containing at least two elements (e.g., thallium bromide or thallium gallium selenide), which were proposed because Si and Ge single detectors need low temperatures to work efficiently at low noise levels [16,17,18].

However, 2D materials such as graphene are emerging as promising materials for solid-state radiation sensors, due to their distinctive electronic properties such as ultra-high electron mobility at room temperature and a bandgap structure that can be easily tailored via external stimuli and doping. Thus, graphene can effectively be used as a radiation sensor by detecting the change in bandgap structure and resistivity due to lattice defects induced by graphene–radiation interactions [19,20]. Furthermore, these defects can be characterized using morphological characterization techniques and changes in the intensity or the location and broadness of specific peaks in the Raman spectrum.

Along these lines, recent research has used graphene in detecting a wide range of radiation dosages from 1 to 20 kGy via physical changes in bandgap characteristics. Gamma-radiation-induced p-doping in graphene affects the graphene’s crystal structure and band properties by breaking the zero-gap property and introducing new vacancies into the lattice structure, affecting electron mobility and thus increasing resistivity and changing the electronic properties of the 2D material [21,22]. In our previous work, graphene was used to enhance the sensitivity of Schottky-junction low-bias radiation sensors by controlling the current passing into the fabricated junction made of n-Si/Pt/graphene and detecting the Schottky barrier’s height and width. The results showed an increase in sensitivity of 11 times compared to the conventional assembly, in parallel with a five-times increase in sensing range, confirming both the validity and benefit of using graphene in such applications [20].

Similarly, integrating monolayer graphene as a radiation-sensing material was reported by Patil et al. [23]. They used a graphene field-effect transistor as a radiation sensor, where local changes in the electrical field due to ionized charges affect the graphene’s electrical properties, which is detected as a signal using the gate voltage between the graphene and the back of the absorber used in the sensor. Their sensor consists of three main stages: a graphene layer over an insulation layer, all bound over a Si layer for radiation absorption. X-rays, gamma rays, and light photons were used to irradiate the sensor, and a 70% change in graphene’s resistance was shown in the case of X-ray irradiation at room temperature. For comparison, smaller resistance responses ranging between 0.5% and 13.6% were measured in SiC-based graphene field-effect transistors in the same dosage range using X-rays. This field-effect technology showed a slow response due to the carriers’ accumulated charges and drift time. In addition, the sensor’s selectivity for irradiation was minimal with respect to the background light photons; thus, it has to operate under dark conditions.

Jain et al. [24] introduced a graphene-based field-effect transistor which was used as a dosimeter, proving that electronic changes in characteristics such as the Dirac voltage and p-doping occurred after gamma irradiation, affecting the mobility of electrons. The back gate was used as a substrate, and a graphene monolayer was transferred onto the substrate, forming the required graphene-based field-effect transistor assembly, which was then irradiated using a Co-60 radiation source with a dosage ranging from 1 kGy to 20 kGy, showing a sensitivity of approximately 1V/kGy. The reported sensitivity originates from the shift in the Dirac point and the electron–hole mobility itself, affecting the electronic gap properties of the material, as proven by Raman spectroscopy. Lower dose ranges were not tested in this study, though they are extremely important for the biomedical and pharmaceutical industries.

The studies mentioned above were mainly aimed at detecting higher doses of ionization energy using different techniques such as monitoring changes in energy and flux, which affect the resistance of graphene, or monitoring changes in drain current in parallel with gate voltage. It was also observed that the defect density could reach around 0.5 × 10^11^ defects/cm^2^ after a radiation dose of 20 kGy. Neither study tested graphene’s sensitivity for smaller irradiation doses below 1 kGy or eliminated the background-light photon cross-sensitivity.

The sensor’s backend readout circuit should be designed to inhibit noise, increase the S/N ratio, enhance the signal quality, and increase sensitivity and selectivity by suppressing any background environmental effects (e.g., light and temperature effects) that may affect the sensor’s reliability. This is particularly important for small-range radiation detectors due to the small changes in resistivity and possible interference from ambient light.

Radiofrequency (RF) oscillators can transform changes in resistance or capacitance into readable frequency signals [25]. LC-tank oscillators [26], crystal oscillators [27], and ring oscillators [28,29,30] are the most commonly used oscillators for sensors’ backend circuits. LC-tank oscillators are widely used for high-frequency-range devices. However, they are severely affected by parasitic effects, with lower control over the amplitude of the oscillations. Crystal oscillators are known for their stable oscillation frequency but with a limited frequency range which is not tunable for wide-range sensors such as radiation sensors. Ring oscillators are mainly used for more minor changes in frequency, with the advantages of low power consumption and greater efficiency in the case of flicker noise and jitter caused by the whole sensor assembly.

Thus, in this work, we propose a monolayer graphene radiation sensor capable of detecting ionizing radiations at low (below 1 kGy) ranges with high sensitivity and resolution. Two identical sensors are connected to a backend RF ring oscillator circuit to minimize the ambient background effects, such as light photons. Incident irradiations cause an accumulated graphene lattice defect density, leading to changes in the bandgap structure and decreasing the electron mobility, leading to increased resistivity, which can be detected as the percentage change in oscillating frequency in the RF ring oscillator readout circuit. Thus, in this paper, we introduce the criteria for transforming the change in graphene resistivity caused by cumulative ionizing radiation into a change in oscillating frequency detected via the oscillating frequency of a backend RF ring oscillator circuit, with no need to monitor gate voltages and drain currents.

## 2. Working Principle

**Figure 1:**
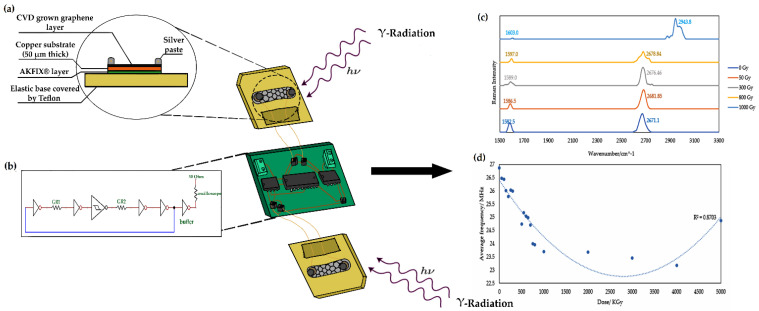
Schematic summarizing the working criteria for the proposed radiation sensor. (**a**) A cross section through the graphene-based sensing part. (**b**) RF ring oscillator circuit design. (**c**) Raman spectra of graphene after irradiation at different doses. (**d**) Average frequency plot for the applied irradiation dose, showing a drop in average frequency after irradiation.

The working principle is based on the induced lattice defects in graphene in response to ionizing radiation. These defects eventually shift the graphene towards a non-crystalline phase [31], in parallel with p-doping due to interaction with air molecules [21,22,32], thus decreasing the electron mobility and increasing the graphene’s resistivity. As shown in Figure 1, two irradiated graphene sensors were connected to the RF ring oscillator transducer circuit to monitor the change in the oscillating frequency in response to the different radiation doses. The oscillating frequency decreases as the resistivity of the graphene increases, generating a change in frequency signal that corresponds to the cumulative irradiation dose. The change in frequency as a result of the change in graphene resistance can be estimated by:

(1) $$\frac{\Delta F}{F}=\frac{\Delta R_{g}}{\gamma +\Delta R_{g}}\;\;,$$

where γ is a complex constant that can be estimated by:

(2) $$\gamma =\frac{R_{on}KN}{M}+R_{g},$$

where $R_{on}$ is the resistance of the inverter when it is in the on mode, K is $\;1+\frac{C_{d}}{C_{g}}$, $R_{g}$ is the original graphene resistance, N is the number of inverter stages, and M is the number of graphene sensors. Using Equation (2), γ was estimated to be 37.7 kΩ.

The benefits of this approach are the high electron mobility of monolayer graphene and the high sensitivity and selectivity towards ionizing radiation. The proposed backend ring oscillator circuit suppresses environmental background effects (e.g., from light photons) by integrating two graphene sensors, as shown in Figure 1a,b, which amplifies the signal caused by the ionizing irradiation over that of the interactions with ambient light photons.

The device consists of two graphene sensors, as shown in Figure 1a, connected to the RF backend circuit by Cu wiring. The backend RF ring oscillator circuit consists of a series of odd-numbered inverters, including a Schmidt trigger, connected in series with the two graphene-based sensing parts. This assembly correlates changes in the resistivity of the graphene due to cumulative irradiation with the corresponding changes in the oscillating frequency of the backend circuit, generating a change-in-frequency signal for each irradiation dose.

The circuit shown in Figure 1b was designed to decrease the jitter in the output frequency and to suppress the effects of ambient light and temperature on the resulting change in oscillating frequency. After each irradiation dose, the graphene sensors were characterized using Raman spectroscopy, as shown in Figure 1c, to characterize the radiation-induced lattice defects and compare them with the change in frequency caused by the increase in the graphene’s resistance, as shown in Figure 1d.

## 3. Materials and Methods

### 3.1. Materials

CVD-grown graphene on a 50-micron copper foil substrate was used in this study as the sensing material and was purchased from 2D Semiconductors (Scottsdale, AZ, USA), where procedures were used to allow graphene layer growth over the whole copper foil surface area without its being affected by the vacancy point defect concentration. The growth of the graphene monolayer required multiple processes, to achieve the required structure with all of the properties needed for our radiation-sensor device.

Copper foil was first heated to elevated temperatures of around 1035 °C, then annealed while ensuring a constant flow of hydrogen gas, before starting a 3 h growth in a constant flow of a gaseous mixture of hydrogen and methane at the above-mentioned temperature. Finally rapid cooling was achieved by opening the furnace. Post-growth processes are usually used, such as cutting and storing in a humid environment for two days to allow oxidation of the copper layer and to weaken any van der Waals forces to ease the detachment of the graphene layer if transfer to another substrate is required. However, in our case, the graphene/Cu interaction was needed [33].

### 3.2. Device Fabrication

Samples of graphene/Cu were mechanically cut into 30 mm^2^ rectangular wafers. Then, these wafers were attached to a fabricated elastic substrate to ensure protection while handling the samples during irradiation. The elastic base consisted of a paper rectangle covered with Kapton tape on both sides, with a surface area of around 25 cm^2^. The wafer was attached to the elastic base using an acrylonitrile AKFIX^®^ binder, to fix the sample without slipping. This binder is used in multiple sensor applications such as strain gauges, eliminating any slipping effect with reliable adhesion. A drop of around 0.1 cm^3^ of the binder was added onto the substrate. The wafer was fixed carefully over the adhesive drop to avoid any of the binder getting onto the sample and damaging the graphene layer.

An electrically conductive silver paste connected the Cu wiring to the graphene surface. The silver paste consisted of 3 main components: the conductive silver particles, representing around 60 wt%, an auxiliary agent, and a solvent for the other two components. Two drops of the pre-cured paste of around 0.15 cm^3^ each were added onto two copper wires, each located at one end of the wafer, as shown in Figure 1, then cured for 16 to 20 h at room temperature. Alternatively, the paste can be cured for 30 min at 120 °C to 200 °C, but heat energy was avoided in this fabrication process [34].

### 3.3. Gamma and Beta Irradiation

Gamma irradiation was applied to the graphene sensors while they were disassembled from the backend circuit. Gamma irradiation was applied using a Co-60 source to achieve cumulative irradiation by subjecting the graphene to the source for specific periods. Similar gamma irradiation was applied to the 2 graphene-based sensors to be subsequently connected to the backend ring oscillator circuit after the irradiation process for monitoring the induced signal via the transducer. Gamma irradiation was applied between 0 to 1 kGy, followed by RF ring oscillator characterization. The irradiation test was repeated with beta radiation for different samples using an electron beam source; the oscillating frequency was recorded using an oscilloscope for a cumulative beta-radiation dose ranging between 0 and 9 kGy.

### 3.4. Raman Spectroscopy

Raman spectroscopy was used as a primary material characterization technique to detect the effects on the graphene lattice that could evolve due to ionizing irradiation, such as the changes in the Dirac point caused by any deformed crystal that will affect the electron mobility through the graphene layer. A dispersive Raman spectrometer produced by Bruker (Billerica, MA USA) with a 20 mW, 532 nm laser source was used to characterize the irradiated samples, using a 50 × 1000 µm aperture.

Unirradiated graphene demonstrated sharp graphene peaks that appeared to be clear and clean, as shown in the Raman analyses in Section 4.2, e.g., the 2D peak with a wavenumber of 2671.11 cm^−1^. The G peak was located at a wavelength of 1582.7 cm^−1^, with an $\frac{I_{2D}}{I_{G}}$ ratio of 1.99. From this value of the ratio, we can conclude that the copper substrate used had a (111) crystal orientation, according to a study by Frank et al. that compared the effect of different crystal orientations of copper substrates with graphene on the surface on the Raman spectra results [35].

### 3.5. SEM Characterization

The morphology of the graphene monolayer was examined and studied using field-emission scanning electron microscopy (FE-SEM, Zeiss SEM Ultra 60) after each irradiation process, using magnifications that ranged between X250 to X1500, with an electron high-tension value of 4 kV and a working distance of 4.9 mm.

## 4. Results and Discussion

### 4.1. Study of Ambient Light Effects

An experiment was conducted on our sensor assembly in order to determine the impact of light photons on the oscillating output frequency generated by the RF ring oscillator circuit under different light conditions, where cumulative gamma-radiation doses ranging from 0.8 kGy to 1 kGy were applied to the two graphene sensors and oscillating frequency readings were recorded from the oscilloscope under three different environmental conditions: (i) with the two graphene films subjected to light, (ii) with one graphene film subjected to light and the other kept in darkness, and (iii) with both graphene films kept in darkness.

**Figure 2:**
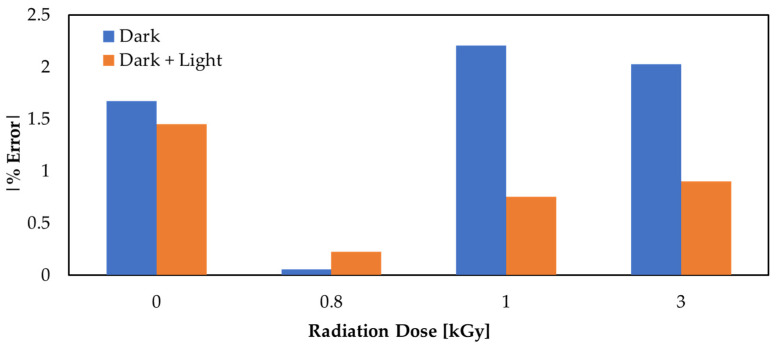
The relationship between % error and dosage in two different light modes.

As shown in Figure 2, the % error of each dose was calculated under two different light conditions and plotted in one figure, using the following equation:

(3) $$\%\;error=\frac{F_{without\;light}-F_{light}}{F_{light}}*100$$

The results showed an average % error of 0.451% in the case of two graphene sensors in darkness and 0.457% in the case of one graphene sensor in light and the other in darkness, proving the ability of the RF ring oscillator backend circuit used to eliminate the background effects, thus proving it has high selectivity with respect to ambient light photons.

Furthermore, uncertainty calculations regarding the ambient light effect were performed using the % error calculations by taking the derivative of Equation (2):

(4) $$\frac{d\Delta Rg}{d\frac{\Delta F}{F}}=\frac{\;\gamma }{{\left(\frac{\Delta F}{F}-1\right)}^{2}}\;,$$

where $\frac{\Delta F}{F}\;$ was experimentally determined, together with its error due to the background light effect, and $\frac{\Delta F}{F}=0.026899537\;\pm 0.0032679151$ at 0.8 kGy. The light error value in $\frac{\Delta F}{F}$ was subsequently substituted into the following equation to calculate the uncertainty in the change in graphene resistance due to background light, $W_{\Delta Rg}$:

(5) $$W_{\Delta Rg}={\left({\left(\frac{d\Delta Rg}{d\frac{\Delta F}{F}}\right)}^{2}*{\left(w_{\frac{\Delta F}{F}}\right)}^{2}\right)}^{0.5}=0.1301058\%\;or\;1.344\;Ω,$$

The uncertainty in $\Delta R_{g}$ was 1.344 Ω, which represents a 0.1301058% difference from the calculated $\Delta R_{g}$ at the irradiation dose of 0.8 kGy.

### 4.2. Raman Characterization

**Figure 3:**
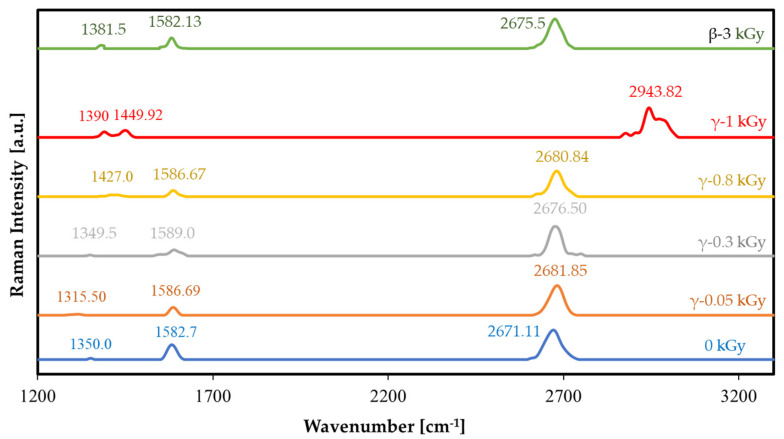
Normalized Raman spectra of CVD-grown graphene on the copper substrate after cumulative gamma irradiation from 0 Gy to 1 kGy.

As shown in Figure 3, to study the vibrational excitations of the graphene bonds resulting from the inelastic scattering of photons, the graphene layers were analyzed by Raman spectroscopy after each gamma-irradiation dose.

Intrinsic (i.e., unirradiated) Raman bands or peaks for graphene were first analyzed, for the D-band at 1350 cm^−1^, the G-band at 1582.7 cm^−1^, and finally the 2D band at 2671.11 cm^−1^. It was observed from the Raman spectra in Figure 3 that the G-band and 2D-band shifts increased after irradiation. As discussed before, this was due to the p-doping effect of the ionizing radiation causing broadening and splitting of the 2D band at higher radiation doses, reflecting the breaking of bonds and the stacking of broken graphene layers.

Of particular interest in this study were the intensities and the Raman shifts of the G-band and comparing these with other doses to detect the changes in electronic properties such as the bandgap. As shown in Table 1, the ratio of $\frac{I_{\;D}}{I_{G}}$ defect densities due to cumulative irradiation was calculated using Equation (6), using the peak values of the Raman spectra shown in Figure 3 after each gamma-irradiation dose ranging between 0 and 1 kGy. This ratio increased as the gamma-irradiation dose increased. The G-band and 2D-band shifts were also observed and compared with the intrinsic peak position of graphene, as shown in Table 1, showing an increase in the shift value with an increase in irradiation dose. The defect density was calculated using the following equation [36]:

(6) $$N_{D}=\left(1.8\pm 0.5*\frac{10^{22}}{\lambda_{L}^{4}}\right)*\left(\frac{I_{D}}{I_{G}}\right)$$

As observed from Table 1, $N_{D}\;$(the defects per ${\mathrm{cm}}^{2}$ of graphene) increased with an increase in cumulative gamma irradiation dosage, due to deformations in the hexagonal crystal structure of graphene in the domains of monolayered graphene, caused by the ionization radiation (and verified via SEM), leading to an increase in defect density which decreases electron mobility, increasing the bandgap of the graphene and causing an increase in resistivity and thus a decrease in the measured oscillating frequency.

It was also observed that the broadness of the 2D peak increased with increasing cumulative radiation dose, resembling the layering effect caused by cracking and stacking of monolayer graphene into multiple layers [37].

Raman spectroscopy was repeated in the same way for beta irradiation, as shown in Figure 3, where 3 kGy of cumulative beta radiation led to a calculated $\frac{I_{\;D}}{I_{G}}$ of 0.683, which is almost equivalent to $0.683\times 10^{11}$ defects per ${\mathrm{cm}}^{2}$. This defect density is lower than that caused by 1 kGy of gamma irradiation, proving that the physical lattice changes caused by beta irradiation of the graphene are much lower than the effects induced by gamma irradiation. This can also be attributed to ozone adsorption caused by beta irradiation without a vacuum, causing a p-doping effect that is lower than that caused by gamma irradiation [32].

### 4.3. Morphological Characterization

**Figure 4:**
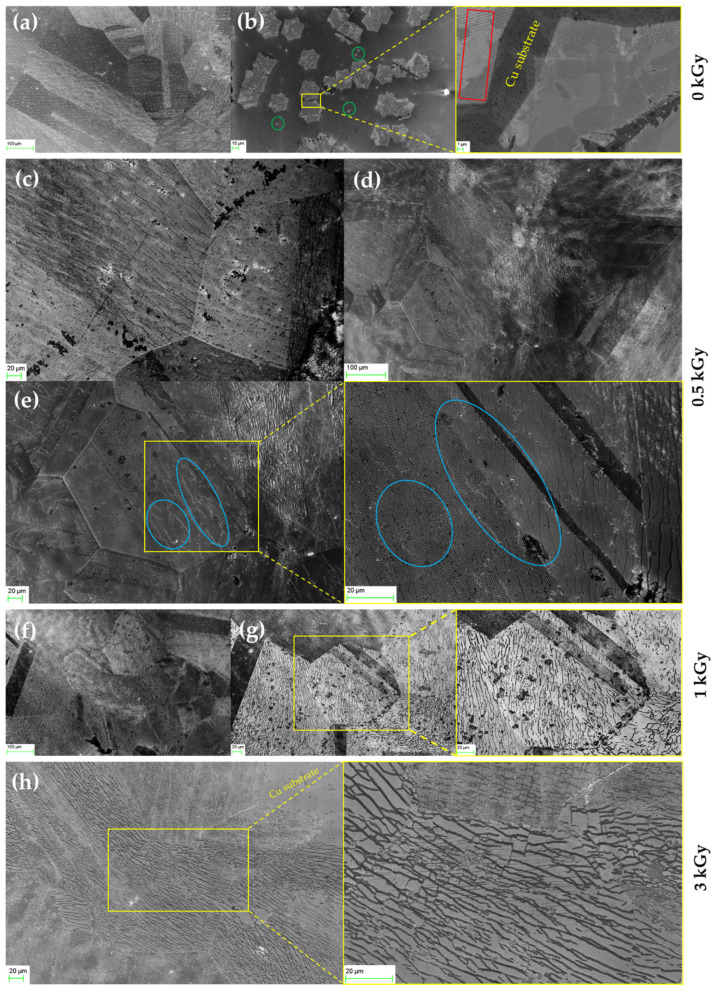
SEM images of graphene (**a**,**b**) before and (**c**–**h**) after gamma irradiation. (**a**,**b**) Before irradiation (graphene nuclei shown in green circles and lattice lines highlighted in red box). (**c**–**e**) After 0.5 kGy irradiation (wrinkles highlighted by blue circles). (**f**,**g**) After 1 kGy irradiation, showing thick crack lines evolved. (**h**) After 3 kGy, showing thicker interconnected crack lines in the graphene domains.

A scanning electron microscope (SEM) was employed to verify the evolution of microstructural defects caused by gamma and beta irradiation observed in the Raman analysis. Figure 4 shows SEM images of the graphene morphology before and after gamma irradiation with different doses ranging between 0.5 and 3 kGy. The SEM images showed that the CVD graphene domains grew in a dendritic pattern. The graphene nuclei are highlighted by green circles in Figure 4b. Lattice lines are also shown in the zoomed SEM image in Figure 4b (highlighted by the red box), with copper substrate surrounding the graphene domains. Figure 4c–e show the morphology of 0.5 kGy gamma-irradiated graphene, clearly showing the evolution of the microstructural defects at the domain level and the increase in defect number, size, and distribution. Figure 4c–e shows the emergence of wrinkles (highlighted by blue circles in Figure 4e) caused by gamma irradiation. 

Wrinkles could be observed as light-grey lines inside the domain and are outlined by blue circles in the zoomed-in Figure 4e. Figure 4f,g shows that these wrinkles further evolve into cracks after 1 kGy of irradiation, verifying the dramatic increase in defect density after 0.8 kGy that was observed in the Raman analysis. The cracks can be observed as dark lines. Beyond 1 kGy of gamma irradiation, the generated cracks developed into denser and thicker channels, as shown in Figure 4h for the 3 kGy irradiated graphene. Figure 4h also shows that these channels became more continuous and interconnected compared to the 1 kGy case.

**Figure 5:**
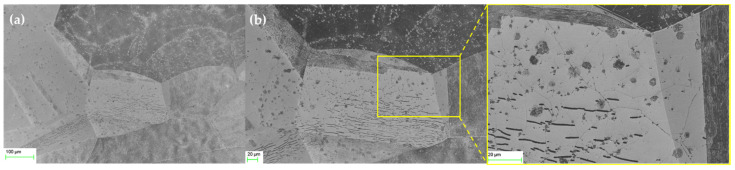
(**a**) SEM images of graphene after 3.5 kGy beta irradiation. (**b**) Zoomed-in image showing induced cracks on graphene after gamma irradiation.

Figure 5 shows an SEM image of the graphene sensor after a beta-irradiation dose of 3.5 kGy, showing the defects induced by beta irradiation. As can be seen in Figure 5, compared with gamma irradiation, beta irradiation affected the graphene less in terms of microstructural defects, manifested by fewer wrinkles and less cracking, as shown in Figure 5b. The observed defects for 3.5 kGy of beta irradiation were lower than for 1 kGy of gamma irradiation (cf. Figure 4f,g), which is in line with the calculated defect densities listed in Table 1.

### 4.4. Backend RF Ring Oscillator Characterization

**Figure 6:**
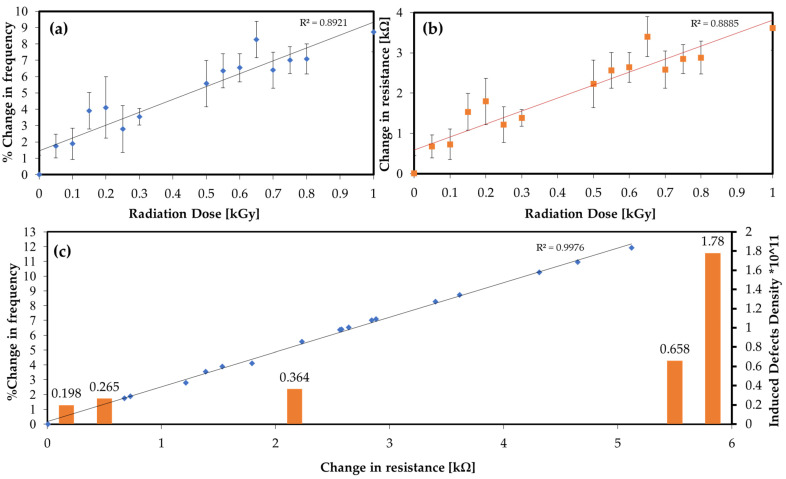
The relation between cumulative gamma-radiation dose with (**a**) % change in frequency and (**b**) change in graphene resistance $\Delta R_{g}$. (**c**) Relation between change in graphene resistance and % change in frequency, showing the corresponding induced defect density.

After each cumulative dose, the irradiated graphene sensors were subsequently connected to the backend circuit to measure the percentage change in the oscillating frequency. Cumulative gamma irradiation was applied between 0.05 Gy and 1 kGy. As shown in Figure 6, an overall linear trend can be fitted in the range up to 1 kGy, with an average sensitivity that can be determined by taking the slope of the curves. This was found to be a 7.86% change in frequency per kGy, calculated from the curve in Figure 6a, which corresponds to a change in resistance $\Delta R_{g}$ of 3.82 kΩ/kGy, calculated from the curve in Figure 6b. It was observed that the standard deviation in the output reading increased rapidly beyond the 1 kGy limit, and the readings began to deviate from linearity at ~1 kGy. This can be correlated with the SEM images and the calculated defect densities in Table 1 for the 1 kGy and the 3 kGy doses (cf. Figure 4f–h and Table 1), which exhibit nonlinear increases in lattice and microstructural defects after 1 kGy. Figure 6c shows the effect of the evolution of lattice defects (expressed as lattice defect density) on the graphene’s resistivity and the output change in frequency.

**Figure 7:**
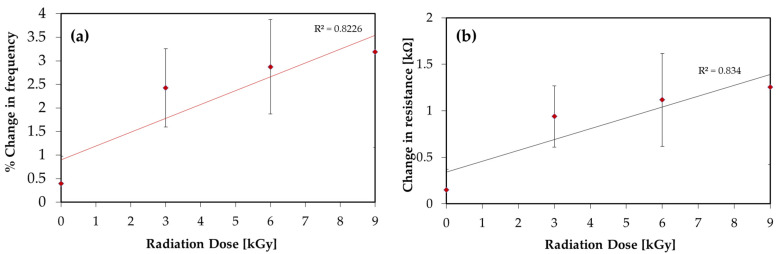
(**a**) The relationships between cumulative beta-radiation dose and % change in frequency, and (**b**) change in resistance, ΔR_g_.

Furthermore, the RF ring oscillator circuit was used to characterize the beta-irradiated (cumulative radiation dose ranging between 0 and 9 kGy) graphene sensors. Figure 7 shows the linear relation between radiation value and % change in frequency, provided the change in frequency results from irradiation in the range between 0 and 9 kGy. The average sensitivity of the sensor to beta irradiation showed a change in R_g_ per Gy of 0.12 kΩ/kGy and % change in frequency per kGy of 0.29%, showing that the sensor was 27.1 times less sensitive than for gamma radiation. This is suggested to be due to the effect of the beta irradiation taking place in an ambient environment, without applying a vacuum, allowing further adsorption of the ozone which is usually located in the electron beam and hence causing a p-doping effect to occur but with lower density than for gamma irradiation, causing a lower change in resistance than for gamma radiation [32].

## 5. Conclusions

The proposed monolayer graphene ionizing radiation sensor platform was tested for cumulative irradiation with two different ionizing radiation types (gamma, and beta), with irradiation ranges of 0–1 kGy for gamma and 0–9 kGy for beta radiation, filling the research gap of sensing in the low-to-medium range of radiation doses (below 1 kGy). Raman spectroscopy and morphological characterizations showed that cumulative irradiation increases the defect density and allows for p-doping, which changes the bandgap properties of graphene, decreasing the mobility of electrons and thus increasing the resistance. The irradiated graphene sensors were further characterized morphologically using an SEM analysis, which further verified the effect of ionizing irradiation on the evolution of defects. The SEM analysis also further verified the more negligible effects of beta irradiation compared to gamma irradiation and the sensor’s linearity limits at ~1 kGy due to the excessive microstructural damage beyond this point. The RF ring oscillator backend circuit transforms the change in frequency into a change in oscillating frequency, to amplify the readout signal for more accurate readings. The circuit was tested for suppression of background light effects, and it was found that the % error due to light reached 0.457%, causing uncertainty in the change in resistance calculation of 1.344 Ω. The irradiation testing results showed a higher sensitivity to cumulative gamma irradiation (3.82 kΩ/Gy) than to beta irradiation (0.12 kΩ/Gy). This can be attributed to the fact that the induced graphene lattice defects and the p-doping due to gamma irradiation were higher than those due to beta irradiation. Due to its low cost, small size, and low power consumption, the proposed sensor is suitable for wireless sensor networks in various applications such as food, agriculture, pharmaceutical, or border security applications.

## Figures and Tables

**Table 1:** Raman spectroscopy analysis of graphene layer at the laser wavelength of 532 nm for gamma-irradiation doses ranging from 0 to 1 kGy and beta-irradiation doses ranging between 0 and 3 kGy.

Type	Irradiation Dose [kGy]	$\frac{I_{\;D}}{I_{G}}$	D-Band Shift $\left[cm^{-1}\right]$	G-Band Shift $\left[cm^{-1}\right]$	2D-Band Shift $\left[cm^{-1}\right]$	$N_{D}\left[\frac{defects}{cm^{2}}\right]\times 10^{11}$
Gamma Irradiation	0	0.088	-	-	-	0.198
	0.05	0.118	−34.5	+3.99	+10.74	0.265
	0.3	0.162	−0.5	+6.30	+5.39	0.364
	0.8	0.293	+77	+3.97	+9.73	0.658
	1	0.792	+40	−132.8	+252.71	1.780
Beta Irradiation	3	0.304	+31.5	−0.57	+4.39	0.683
